# *Mycobacterium kansasii* Infection in a Farmed White-Tailed Deer (*Odocoileus virginianus*) in Florida, USA

**DOI:** 10.3390/ani14101511

**Published:** 2024-05-20

**Authors:** Sydney L. Cottingham, An-Chi Cheng, Pedro H. de Oliveira Viadanna, Kuttichantran Subramaniam, William F. Craft, Marley E. Iredale, Samantha M. Wisely, Juan M. Campos Krauer

**Affiliations:** 1 Department of Large Animal Clinical Sciences, College of Veterinary Medicine, University of Florida, Gainesville, FL 32611, USA; s.cottingham@ufl.edu (S.L.C.); anchicheng@ufl.edu (A.-C.C.); 2 Department of Infectious Diseases and Immunology, College of Veterinary Medicine, University of Florida, Gainesville, FL 32611, USA; pedro.viadanna@wsu.edu (P.H.d.O.V.); kuttichantran@ufl.edu (K.S.); 3 Emerging Pathogens Institute, University of Florida, Gainesville, FL 32611, USA; wisely@ufl.edu; 4 Department of Comparative, Diagnostic, and Population Medicine, College of Veterinary Medicine, University of Florida, Gainesville, FL 32611, USA; craftw@ufl.edu (W.F.C.); marley.iredale@ufl.edu (M.E.I.); 5 Department of Wildlife Ecology and Conservation, University of Florida, Gainesville, FL 32611, USA

**Keywords:** non-tuberculous *Mycobacterium*, pneumonia, captive cervid, deer farming

## Abstract

**Simple Summary:**

*Mycobacterium kansasii* is a zoonotic bacterial pathogen that can cause a chronic disease that resembles pulmonary tuberculosis in many mammals including humans, cattle, goats, black-tailed deer, white-tailed deer, Florida manatees, and rhesus monkeys. We identified the first case of *M. kansasii* infection in farmed white-tailed deer (*Odocoileus virginianus*) in Florida. We performed hematoxylin and eosin (H&E) staining, Ziehl–Neelsen (ZN) staining, PCR, and Sanger sequencing of the lung tissue after the animal was euthanized. The microscopic observations of the H&E-stained lung showed multifocal granuloma, while the ZN-stained tissue revealed low numbers of beaded, magenta-staining rod bacteria inside the granuloma formation. Molecular examination identified the presence of *Mycobacterium kansasii*. Although this was a sporadic case and there was no evidence that *M. kansasii* could be transmitted directly from animals to human beings, it is crucial to have basic biosecurity practices and management in place to prevent disease outbreaks and spillover.

**Abstract:**

A 7-year-old farmed white-tailed deer doe was transported to a Levy County, Florida property and began to decline in health, exhibiting weight loss and pelvic limb weakness. The doe prematurely delivered live twin fawns, both of which later died. The doe was treated with corticosteroids, antibiotics, gastric cytoprotectants, and B vitamins but showed no improvement. The doe was euthanized, and a post mortem examination was performed under the University of Florida’s Cervidae Health Research Initiative. We collected lung tissue after the animal was euthanized and performed histological evaluation, using H&E and Ziehl–Neelsen (ZN) staining, and molecular evaluation, using conventional PCR, followed by Sanger sequencing. The microscopic observations of the H&E-stained lung showed multifocal granuloma, while the ZN-stained tissue revealed low numbers of beaded, magenta-staining rod bacteria inside the granuloma formation. Molecular analysis identified the presence of *Mycobacterium kansasii*. This isolation of a non-tuberculous *Mycobacterium* in a white-tailed deer emphasizes the importance of specific pathogen identification in cases of tuberculosis-like disease in farmed and free-ranging cervids. We report the first case of *M. kansasii* infection in a farmed white-tailed deer (*Odocoileus virginianus*) in Florida. Although *M. kansasii* cases are sporadic in white-tailed deer, it is important to maintain farm biosecurity and prevent farmed cervids from contacting wildlife to prevent disease transmission.

## 1. Introduction

*Mycobacterium kansasii* is a zoonotic pathogen capable of causing chronic, debilitating disease that resembles pulmonary tuberculosis. *M. kansasii* belongs to the phylum *Actinomycetota* and the family *Mycobacteriaceae* [1,2,3]. *M*. *kansasii* is an aerobic, Gram-positive, slow-growing, and photochromogenic species with a genome size of 6.4 Mb [4]. *M. kansasii* is classified as a non-tuberculous mycobacterium (NTM), distinguishing this species from the *Mycobacterium tuberculosis* complex (MTBC) [4]. *M. kansasii* is ubiquitous in the environment worldwide, particularly in soil and tap water [3,5].

*M. kansasii* was first described as an opportunistic pathogen in human patients with pulmonary disease resembling tuberculosis in Kansas, USA in the 1950s [6]. Clinical signs of *M. kansasii* in humans include anorexia, progressive weight loss, weakness, low-grade intermittent fever, and respiratory signs [7]. *M. kansasii* is more common in immunocompromised patients [8,9,10,11] and patients with existing pulmonary conditions, such as chronic obstructive pulmonary disease [12,13]. Human-to-human transmission of *M. kansasii* has not been confirmed [4,14].

*M. kansasii* is ubiquitous in the environment, yet is a rare pathogen in livestock and wildlife [4]. The clinical presentation of *M. kansasii* infection in animal hosts is highly variable, ranging from asymptomatic infection to overt clinical disease that may feature chronic weight loss, granulomatous pneumonia, bronchial lymphadenopathy, or cutaneous lesions [15,16,17,18,19,20,21,22,23,24,25,26,27,28]. Disease associated with *M. kansasii* infection has been reported in wild boar (*Sus scrofa*) [27], free-ranging black-tailed deer (*Odocoileus hemionus columbianus*) [17] and white-tailed deer (*Odocoileus virginianus*) [25], bonteboks (*Damaliscus pygargus dorcas*) [20], laboratory rhesus monkeys (*Macaca mulatta*) [22], captive Florida manatees (*Trichechus manatus*) [16], domestic goats (*Capra hircus*) [15], cattle (*Bos taurus*) [19,23,24,28], domestic cats (*Felis catus*) [26], Sichuan takins (*Budorcas taxicolortibetana*) [21], siamangs (*Hylobates syndactylus*) [21], and alpacas (*Vicugna pacos*) [18]. Herein, we report the first known case of *M. kansasii* infection in a farmed white-tailed deer.

## 2. Materials and Methods

In March 2018, an approximately 7-year-old farmed white-tailed deer doe was transported to a private property in Levy County, Florida. The owner of the doe reported progressive weight loss and pelvic limb weakness but did not initiate treatment as the doe was believed to be pregnant. The doe eventually delivered premature twin fawns, both of which died after birth. The owner administered a corticosteroid (intramuscular (IM), dexamethasone) at dosages varying between 4 and 12 mg daily for 28 days, a macrolide antibiotic (1.5 cc, IM, tylosin, Tylan 200 Injection, Elanco US, Inc., Greenfield, IN, USA), a gastric cytoprotectant (1 scoop, orally, sucralfate, Carafate©, Aspen Pharmacare, Durban, South Africa), supplemented B vitamin complex, vitamin B12, and thiamine at unknown dosages. Due to the lack of response to treatment, the doe was euthanized by the owner in August 2018. The University of Florida’s Cervidae Health Research Initiative (CHeRI) was contacted to request a post mortem examination.

**Figure 1:**
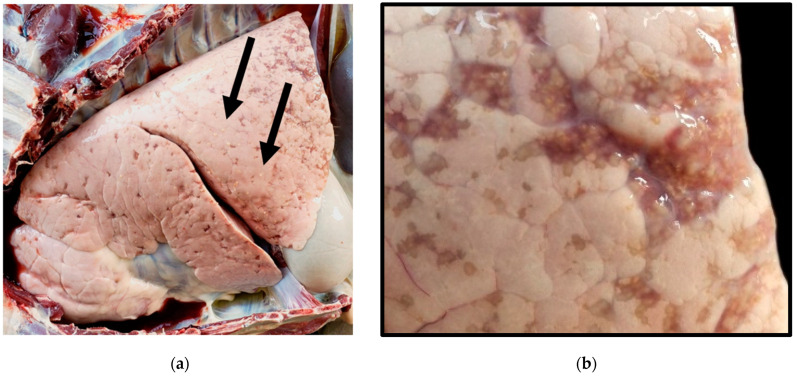
Gross appearance of the lungs in the *M. kansasii*-infected deer. (**a**) Open thoracic cavity demonstrating lungs with multifocal, granulomatous pneumonia. Arrows indicate granulomas. (**b**) Focal area of atelectasis with numerous granulomas.

At necropsy, the doe presented in poor nutritional condition with a dull, rough-hair coat. The lungs were pale, with multifocal, 0.2–0.5 cm yellow-white-to-gray, granular, nodular lesions distributed across the pleural surface and throughout the parenchyma (Figure 1). The greatest density of nodular lesions was observed in the cranial lobes bilaterally. Paired lung, heart, liver, kidney, and spleen samples were chilled at 4 °C and fixed in 10% neutral buffered formalin. Fresh and fixed lung tissues were submitted to the University of Florida College of Veterinary Medicine Diagnostic Laboratories for histopathological and microbiological characterization.

### Molecular Examination

DNA from the lung and isolated bacteria was extracted using DNeasy Blood and Tissue Kit (Qiagen, Hilden, Germany) according to the manufacturer’s instructions. Polymerase chain reaction (PCR) for *Mycobacterium* spp. was carried out using the primer pair KY18-KY75, which amplified a fragment (~500 bp) of the 16S rRNA [25]. Total reaction volumes were 30 µL, which consisted of 0.15 µL of Platinum Taq DNA Polymerase (Invitrogen), 3.0 µL of 10× PCR Buffer, 1.2 µL of 50 mM MgCl2, 0.6 µL of 10 mM dNTPs, 1.5 µL of 20 µM of each forward and reverse primer, 17.6 µL of molecular-grade water, and 4.5 µL of DNA template (100 ng). An initial denaturation step of 95 °C for 5 min was followed by 40 cycles of a 95 °C denaturation step for 5 s, a 55 °C annealing step for 5 s, a 68 °C extension step for 25 s, and a final extension step at 71 °C for 1 min. PCR products were subjected to gel electrophoresis using a 1% agarose gel stained with ethidium bromide. Amplicons of the expected size were purified using a QIAquick PCR Purification Kit (Qiagen). The concentration of purified amplicons was determined fluorometrically using a Qubit 3.0 Fluorometer and dsDNA BR Assay Kit (Life Technologies, Carlsbad, CA, USA) before submitting to Functional Biosciences, Inc. (Madison, WI, USA) for Sanger sequencing. The sequences were visualized and assembled using BioEdit 7.1.3.0.

## 3. Results

### 3.1. Microscopic Observations

**Figure 2:**
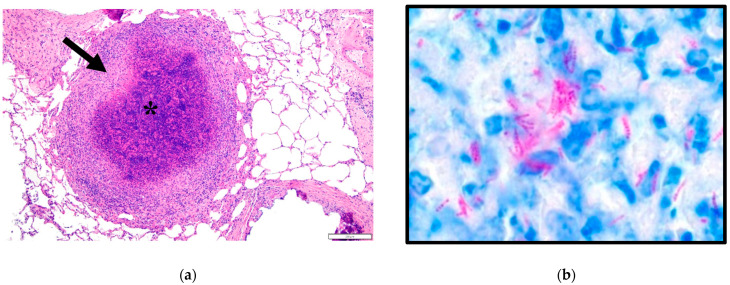
Microscopic observations of lung tissue from the *M. kansasii*-infected deer. (**a**) H&E-stained histologic section of lung with a focal granuloma (bar = 200 µm); asterisk indicates necrotic center. Arrow indicates fibrous capsule with macrophages. (**b**) Ziehl–Neelsen acid-fast stain with low numbers of beaded, magenta-staining rod bacteria (bar = 10 µm).

Microscopically, the lung architecture was effaced by multifocal small-to-large granulomas with central areas of necrosis varying from caseous to lytic, with mild to prominent mineralization within necrotic centers (Figure 2). The necrotic centers were bordered by peripheral rims of epithelioid macrophages that often had finely vacuolated cytoplasm, low numbers of Langhans-type multinucleated giant cells, and scattered lymphocytes, plasma cells, and neutrophils. The granulomas were bordered by variable degrees of fibrosis, with more prominent fibrosis around granulomas with prominent central mineralization (likely indicating chronicity). The alveolar septa were mildly to moderately expanded by lymphocytes, plasma cells, macrophages, several neutrophils, small amounts of fibrin, and congested capillaries (interpreted as interstitial pneumonia). Ziehl–Neelsen staining revealed low numbers of approximately 5–15 μm long, occasionally beaded, slender, acid-fast rods that were extracellular and within macrophages and Langhans-type multinucleated giant cells. Significant lesions were not detected in the heart, spleen, liver, and kidney. The presence of acid-fast rods within granulomas with caseous necrosis and mineralization was highly suggestive of infection with tuberculoid *Mycobacterium* spp. and prompted the molecular identification of the bacteria.

### 3.2. Molecular Examination

A *Mycobacterium*-screening culture (Lowenstein–Jensen agar slants at 35–37 °C and 5% CO_2_, and Middlebrook 7H11 agar at room temperature without added CO_2_) isolated a rapid-growing *Mycobacterium* sp. at around 7 days after culturing. A collected colony and a fragment of the lung were tested for *Mycobacterium* using a specific PCR assay [17] followed by Sanger sequencing. The obtained sequence was 375 bp. A standard nucleotide–nucleotide BLAST (BLASTN) search revealed 100% query coverage and 99.48% identity to 79 strains/isolates of *Mycobacterium kansasii* within the National Center for Biotechnology Information GenBank database. *M. kansasii* is considered a slow-growing *Mycobacterium* species; however, PCR and Sanger sequencing conclusively confirmed the presence of this bacterium. Aerobic culture (general blood agar, chocolate agar, Columbia CNA agar, and MacConkey agar at 35–37 °C and 5% CO_2_) of lung tissue found no growth at 24 h and very scant growth of mixed bacterial and fungal flora, without any clear predominance of the four morphotypes observed after an extended culture period.

## 4. Discussion

We report the first known isolation of *M. kansasii* from a farmed white-tailed deer. The histopathological changes observed in this case are consistent with lesions described in *M. kansasii* infections reported in a hunter-harvested, free-ranging black-tailed deer in Washington [17] and a free-ranging white-tailed deer in Louisiana [25]. *M. kansasii* more commonly infects immunocompromised individuals. Corticosteroids have known immunosuppressive activity, and oral dexamethasone is associated with the reactivation of latent *Mycobacterium* spp. infections in HIV patients. In this case, the history of extended corticosteroid therapy and transportation to a new facility may have produced an immunocompromised state that promoted the reactivation of a latent *M. kansasii* infection. No comorbidities were detected via post mortem examination or ancillary diagnostics, suggesting that *M. kansasii* was the primary cause of death. Though aerobic culture found bacterial and fungal flora in cultured lung tissue, microbial growth was scant, and no known pathogens were isolated.

## 5. Conclusions

This case highlights the importance of positively identifying mycobacterium species associated with tuberculous disease in captive or managed wildlife and underscores the risk of introducing disease agents when translocating livestock and captive wildlife. Mycobacteriosis associated with *M. kansasii* is not a reportable disease; however, bovine tuberculosis (bTB) caused by *Mycobacterium bovis* is a reportable zoonotic agent, and the diagnosis of bTB in captive and free-ranging wildlife has significant regulatory and public health implications for both livestock producers and wildlife managers. Identifying the *Mycobacteria* species associated with tuberculosis-like disease in captive wildlife is critical in mitigating risks to domestic livestock and free-ranging wildlife populations. 

Despite *M. kansasii* not being a reportable disease, the pathogen still poses a risk as an opportunistic pathogen in immunocompromised humans. Deer farming brings humans into close contact with captive cervids, and this increased interaction between farmed cervids and human caretakers elevates the risk of transmission of potential zoonotic pathogens, including *M. kansasii*. It is crucial to maintain basic biosecurity and hygiene practices to reduce the risk of zoonotic disease transmission.

## Data Availability

Data are contained within the article.
